# COVID-19 symptoms, duration, and prevalence among healthcare workers in the New York metropolitan area

**DOI:** 10.1017/ice.2020.1334

**Published:** 2020-11-20

**Authors:** Fran A. Ganz-Lord, Kathryn R. Segal, Michael L. Rinke

**Affiliations:** 1 Department of Medicine, Montefiore Medical Center, Bronx, New York; 2 Occupational Health Services COVID-19 Response, Montefiore Medical Center, Bronx, New York; 3 Albert Einstein College of Medicine, Bronx, New York; 4 Department of Pediatrics, Children’s Hospital at Montefiore, Bronx, New York

## Abstract

**Objective:**

To evaluate symptoms, workforce implications, and testing patterns related to the coronavirus disease 2019 (COVID-19) pandemic among healthcare workers (HCWs) in the New York metropolitan area during spring 2020.

**Design:**

Retrospective cohort study of occupational health services (OHS) records.

**Setting:**

A large, urban, academic medical center with 5 inpatient campuses and multiple ambulatory centers throughout Bronx and Westchester counties.

**Participants:**

We included HCWs who called OHS to report COVID-19 symptoms and had either severe acute respiratory syndrome coronavirus 2 (SARS-CoV-2) polymerase chain reaction (PCR) or IgG antibody testing.

**Methods:**

We analyzed the impact of COVID-19–related symptoms on (1) time from symptom onset to return to work, (2) the results of SARS-CoV-2 nasopharyngeal PCR testing, and (3) the results of SARS-CoV-2 IgG antibody testing in HCWs with mild-to-moderate COVID-19.

**Results:**

The median time from symptom onset until return to work for HCWs who did not require hospitalization was 15 days (interquartile range, 10–22). Shortness of breath, fever, sore throat, and diarrhea were significantly associated with longer durations from symptom onset to return to work. Among symptomatic HCWs who had PCR testing during the study period, 51.9% tested positive. Of the previously symptomatic HCWs who had IgG antibody testing, 55.4% had reactive tests. Ageusia was associated with having both positive PCR and reactive antibody tests. Sore throat was associated with both negative PCR and nonreactive antibody tests.

**Conclusion:**

HCWs with COVID-19 who did not require hospitalization still had prolonged illness. Shortness of breath, fever, sore throat, and diarrhea are associated with longer durations of time away from work.

The severe acute respiratory syndrome coronavirus 2 virus (SARS-CoV-2) has rapidly spread throughout the world. As of August 2, 2020, there were 416,843 confirmed cases of coronavirus disease 2019 (COVID-19) in New York state.^1^ On March 23, 2020, Governor Andrew Cuomo issued an order that required all hospitals in New York to increase capacity by at least 50%.^2^ This order placed an unprecedented requirement on hospitals and healthcare workers (HCWs), who themselves faced high incidences of illness from exposure in both the workplace and the community. Furthermore, early in the pandemic when testing supplies were scarce, HCWs had greater access to testing than the general population. Both of these factors make HCWs a useful cohort in whom to study the emergence and manifestations of and immune response to SARS-CoV-2.

Research on COVID-19 is emerging, but little is known about the relationship between specific symptoms and illness duration. Studies that focus on duration of illness are limited to inpatient cohorts, in whom digestive symptoms have been shown to prolong illness.^3,4^ Meanwhile, outpatients with milder disease can have extended illness duration.^5^ During spring 2020, illness-related work absences rose nearly 5-fold compared to 2019 through all sectors of the United States economy.^6^ Thus, research is needed to identify the impact of COVID-19 on disruptions to the labor force. In particular, analyzing the experience of HCWs with COVID-19 can help hospitals more accurately predict when ill staff may become available to return to work.

The relationship between symptomology and the results of the SARS-CoV-2 nasopharyngeal polymerase chain reaction (PCR) and immunoglobulin G (IgG) antibody tests are still being elucidated. Anosmia and ageusia have been the most consistent indicators of PCR positivity and seroconversion.^7–15^ Some studies report high false-negative rates with PCR testing,^16–18^ and others suggest that PCR positivity does not always predict antibody seroconversion.^12^ Because symptoms of COVID-19 overlap with those of other illnesses and considering the limitations of SARS-CoV-2 testing, it is also important to understand the relationship between PCR and antibody results. Demonstrating a high concordance would confer greater confidence in the diagnosis.

We conducted an observational study of the relationship between COVID-19 symptoms and illness duration during a period of high COVID-19 prevalence. We also examined the relationships between symptoms of COVID-19 and both PCR and IgG antibody test results, as well as between PCR positivity and IgG antibody detection.

## Methods

### Setting

This retrospective cohort study was conducted among HCWs at Montefiore Medical Center (MMC). MMC is a large, urban, academic medical center with 5 inpatient campuses and multiple ambulatory centers throughout Bronx and Westchester counties, 2 of the hardest hit counties in the United States during the study period.^19^ MMC is the largest healthcare provider in the Bronx, which has shown the highest rates of infection, hospitalization, and fatality due to COVID-19 in New York City.^20^

### Study population and data management

The study population consisted of all HCWs who called the Occupational Health Services (OHS) office between March 1 and June 12, 2020. This cohort included employees in both clinical and nonclinical roles. HCWs who required hospitalization for COVID-19 were excluded. HCWs called OHS to report potential symptoms of COVID-19, to receive guidance regarding fitness to work and returning to work, and to obtain both PCR and IgG antibody testing. Information about symptoms, dates away from work, and test results were tracked in a REDCap database at the time of calls between HCWs and the OHS (Vanderbilt University, Nashville, TN).^21^ PCR testing was initially limited due to swab and reagent availability, but it became available to all symptomatic HCWs by mid-April. IgG antibody testing was offered to all HCWs 21 days after symptom onset as of late April. Results of tests performed by the MMC laboratory were obtained from electronic medical records. Results of tests performed by non-MMC laboratories were reported to OHS verbally by the HCWs and were entered into the database. All antibody tests were performed at the MMC laboratory.

Notably, guidelines issued by the Centers for Disease Control and Prevention (CDC) and New York State Department of Health (NYSDOH) changed as knowledge of COVID-19 evolved. The REDCap database discretely captured only those symptoms listed by the CDC and NYSDOH. Over time, the number of symptoms captured changed from 4 to 12. The dates on which symptoms were added to the symptom list are included in the footnotes of Tables 1–3.

**Table 1.** Symptoms Associated With Illness Duration

	Frequency	Illness Duration, d		Results of Wilcoxon Rank-Sum Tests^a^		Results of Multivariable Tobit Regression Model^b^		
	(N=1,698),	With Symptom	Without Symptom					
Risk Factor	N (%)	Median (IQR)	Median (IQR)	*z*	*P>|z|*	Coef. (SE)	95% CI	*P*>|t|
**Age, y**								
<31	303 (17.8)					Ref	Ref	Ref
31–40	467 (27.5)					§	§	§
41–50	370 (21.8)					1.97 (0.93)	0.15–3.78	**.034**
51–60	408 (24.0)					3.64 (0.92)	1.83–5.45	**<.001**
61+	150 (8.8)					3.52 (1.24)	1.09–5.96	**.005**
**Symptoms**								
Fever (≥37.8°C)	1063 (62.6)	16 (11–24)	14 (9–21)	−6.20	**<.001**	2.94 (0.74)	1.48–4.40	**<.001**
Cough	1369 (80.6)	16 (11–23)	12 (8–18)	−6.71	**<.001**	§	§	§
Shortness of breath	693 (40.8)	19 (13–28)	14 (9–19)	−11.82	**<.001**	5.64 (0.73)	4.21–7.07	**<.001**
Sore throat	707 (41.6)	17 (11–24)	15 (10–21)	−4.30	**<.001**	2.25 (0.73)	0.82–3.69	**.002**
Diarrhea^c^	356 (36.7)	17 (13–24)	14 (9–21)	−5.49	**<.001**	2.14 (0.76)	0.65–3.63	**.005**
Anosmia^d^	233 (39.3)	15 (10–21)	15 (11–23)	0.90	.37			
Severe muscle aches^e^	97 (53.3)	19 (14–22)	14 (10–25)	−1.77	.076			
Severe fatigue^e^	83 (45.6)	19 (14–22)	14 (10–25)	−1.69	.091			
Persistent or unusual headache^e^	103 (56.6)	18 (12–22)	15 (10–25)	−0.48	.63			
Upper respiratory symptoms^e^	47 (25.8)	18 (12–21)	17 (11–25)	0.24	.81			
Chest pressure^e^	54 (29.7)	19 (13–23)	15.5 (11–24)	−0.99	.32			
Ageusia^e^	64 (35.2)	18 (11.5–21)	16 (11–25)	0.50	.62			
Chills^f^	28 (34.6)	16.5 (13–21)	15 (10–29)	0.18	.86			

Note. IQR, interquartile range; SE, standard error; CI, confidence interval. Study population: healthcare workers (HCWs) who reported at least one symptom and who had a positive PCR test within −2 and +30 days of symptom onset and were cleared to return to work by OHS from March 1 to June 1, 2020.

a Wilcoxon rank-sum tests included employees who called OHS on or after the date on which the symptom began to be discretely captured.

b The results of the multivariable tobit regression model with stepwise backwards regression (N=971).

c Symptom discretely captured beginning March 31, N=971.

d Symptom discretely captured beginning April 6, N=593.

e Symptom discretely captured beginning April 17, N=182.

f Symptom discretely captured beginning April 27, N=81.

§ Variable eliminated in the backwards regression of this model.

**Table 2.** Symptoms Associated With Positive PCR Result

	Total	Positive	Negative	Results of Bivariate Logistic Regression Models^a^		Results of Multivariable Logistic Regression Model^b^	
	(N=3,971)	(N=2,059)	(N=1,912)				
Symptoms	No. (%)	No. (%)	No. (%)	OR (95% CI)	*P* Value	OR (95% CI)	*P* Value
Fever (≥ 100°F)	1971 (49.6)	1287 (62.5)	684 (35.8)	2.99 (2.63–3.41)	**<.001**	1.80 (1.33–2.45)	**<.001**
Cough	2797 (70.4)	1652 (80.2)	1145 (59.9)	2.72 (2.36–3.13)	**<.001**	1.93 (1.43–2.62)	**<.001**
Shortness of breath	1516 (38.2)	860 (41.8)	656 (34.3)	1.37 (1.21–1.56)	**<.001**	§	§
Sore throat	1818 (45.8)	848 (41.2)	970 (50.7)	0.68 (0.60–0.77)	**<.001**	0.53 (0.39–0.72)	**<.001**
Diarrhea^c^	921 (33.1)	437 (36.2)	484 (30.7)	1.28 (1.09–1.50)	**.002**	0.67 (0.48–0.95)	**.023**
Anosmia^d^	408 (19.9)	285 (37.1)	123 (9.6)	5.57 (4.39–7.05)	**<.001**	3.17 (2.05–4.91)	**<.001**
Severe muscle aches^e^	457 (41.7)	130 (44.8)	327 (40.6)	1.19 (0.91–1.56)	.21		
Severe fatigue^e^	456 (41.6)	120 (41.4)	336 (41.7)	0.99 (0.75–1.29)	.92		
Persistent or unusual headache^e^	499 (45.6)	131 (45.2)	368 (45.7)	0.98 (0.75–1.28)	.87		
Upper respiratory symptoms^e^	229 (20.9)	60 (20.7)	169 (21.0)	0.98 (0.71–1.37)	.91		
Chest pressure^e^	266 (24.3)	79 (27.2)	187 (23.2)	1.24 (0.91–1.68)	.17		
Ageusia^e^	187 (17.1)	98 (33.8)	89 (11.1)	4.11 (2.96–5.70)	**<.001**	2.34 (1.56–3.51)	**<.001**
Chills^f^	205 (27.9)	38 (24.7)	167 (28.7)	0.81 (0.54–1.22)	.32		

Note. PCR, polymerase chain reaction; OR, odds ration; CI, confidence interval. Study population: healthcare workers (HCWs) who reported at least 1 symptom and who had a PCR test within −2 and +30 days of symptom onset from March 1 to June 12, 2020.

a Bivariate logistic regressions included employees who called OHS on or after the date on which the symptom began to be discretely captured.

b The results of the multivariable logistic regression model with stepwise backwards (N=1,095).

c Symptom discretely captured beginning March 31, N=2786 (1209 positive, 1,577 negative).

d Symptom discretely captured beginning April 6, N=2055 (769 positive, 1,286 negative).

e Symptom discretely captured beginning April 17, N=1095 (290 positive, 805 negative).

f Symptom discretely captured beginning April 27, N=735 (154 positive, 581 negative).

§ Variable eliminated in the backwards regression of this model.

**Table 3.** Symptoms Associated With Reactive IgG Antibody Result

	Total	Reactive	Nonreactive	Results of Bivariate Logistic Regression Models^a^		Results of Multivariable Logistic Regression Model^b^	
	(N=1,472),	(N=815),	(N=657),				
Symptoms	No. (%)	No. (%)	No. (%)	OR (95% CI)	*P* Value	OR (95% CI)	*P* Value
Fever (≥ 100°F)	725 (49.3)	490 (60.1)	235 (35.8)	2.71 (2.19–3.35)	**<.001**	§	§
Cough	1056 (71.7)	637 (78.2)	419 (63.8)	2.03 (1.62–2.56)	**<.001**	§	§
Shortness of breath	496 (33.7)	287 (35.2)	209 (31.8)	1.17 (0.94–1.45)	.17		
Sore throat	653 (44.4)	326 (40.0)	327 (49.8)	0.67 (0.55–0.83)	**<.001**	0.49 (0.30–0.81)	**.005**
Diarrhea^c^	285 (30.4)	151 (32.4)	134 (28.3)	1.21 (0.92–1.60)	.18		
Anosmia^d^	123 (19.5)	86 (30.8)	37 (10.5)	3.81 (2.49–5.82)	**<.001**	§	§
Severe muscle aches^e^	135 (41.5)	52 (43.3)	83 (40.5)	1.12 (0.71–1.77)	.62		
Severe fatigue^e^	107 (32.9)	36 (30.0)	71 (34.6)	0.81 (0.50–1.31)	.39		
Persistent or unusual headache^e^	125 (38.5)	46 (38.3)	79 (38.5)	0.99 (0.62–1.58)	.97		
Upper respiratory symptoms^e^	67 (20.6)	21 (17.5)	46 (22.4)	0.73 (0.41–1.30)	.29		
Chest pressure^e^	72 (22.2)	24 (20.0)	48 (23.4)	0.82 (0.47–1.42)	.48		
Ageusia^e^	57 (17.5)	35 (29.2)	22 (10.7)	3.43 (1.89–6.19)	**<.001**	3.81 (2.07–7.00)	**<.001**
Chills^f^	48 (21.1)	15 (20.3)	33 (21.4)	0.93 (0.47–1.85)	.84		

Note. IgG, Immunoglobulin G; OR, odds ratio; CI, confidence interval. Study population: healthcare workers (HCWs) who reported at least one symptom and who had an IgG antibody test after symptom onset from March 1 to June 12, 2020.

a Bivariate logistic regressions included employees who called OHS on or after the date on which the symptom began to be discretely captured.

b The results of the multivariable logistic regression model with stepwise backwards (N=325).

c Symptom discretely captured beginning March 31, N=939 (466 reactive, 473 nonreactive).

d Symptom discretely captured beginning April 6, N=632 (279 reactive, 353 nonreactive).

e Symptom discretely captured beginning April 17, N=325 (120 reactive, 205 nonreactive).

f Symptom discretely captured beginning April 27, N=228 (74 reactive, 154 nonreactive).

§ Variable eliminated in the backwards regression of this model.

Illness duration was defined as the number of days between symptom onset and when the HCW was cleared by OHS to return to work. Throughout the study period, the minimum duration of self-isolation was 7 days. HCWs were cleared to return to work if it had been 7 days from the onset of symptoms, if they had been afebrile for 72 hours without antipyretics, and if their symptoms had substantially improved. Only HCWs with a positive PCR test within 1 month of symptom onset were included in the duration analysis. The duration analysis was also limited to HCWs cleared to return to work by June 1, 2020, because the NYSDOH changed the minimum duration of isolation for HCWs from 7 to 10 days on that date.^22^

On June 12, 2020, all data were exported from the REDCap database for analysis.

### Analysis

Wilcoxon rank-sum tests were used to identify associations between symptoms and illness duration. Symptoms significantly associated with illness duration (*P* < .05) were then used in a multivariable tobit regression model, followed by backward variable selection (*P* < .05) adjusted for age. Because all symptomatic HCWs were required to isolate for a minimum of 7 days, a tobit model accounted for the potential left censorship.

Likewise, χ^2^ tests were used to identify associations between symptoms and positive PCR tests, and separately, reactive IgG antibody tests. Symptoms significantly associated with either test in these bivariate analyses (*P* < .05) were included in 2 separate multivariable logistic regression models, followed by backward variable selection (*P* < .05) to identify the symptoms associated with positive PCR or reactive IgG antibody tests.

Lastly, we examined the frequencies of the PCR and IgG antibody test results. In this analysis, we included HCWs who had had both tests done, with the IgG antibody test having taken place at any point after the PCR test.

This study was approved by the Montefiore/Einstein Institutional Review Board. All analyses were performed using Stata version 11.2 software (StataCorp, College Station, TX).

## Results

### Illness duration analysis

**Fig. 1.**
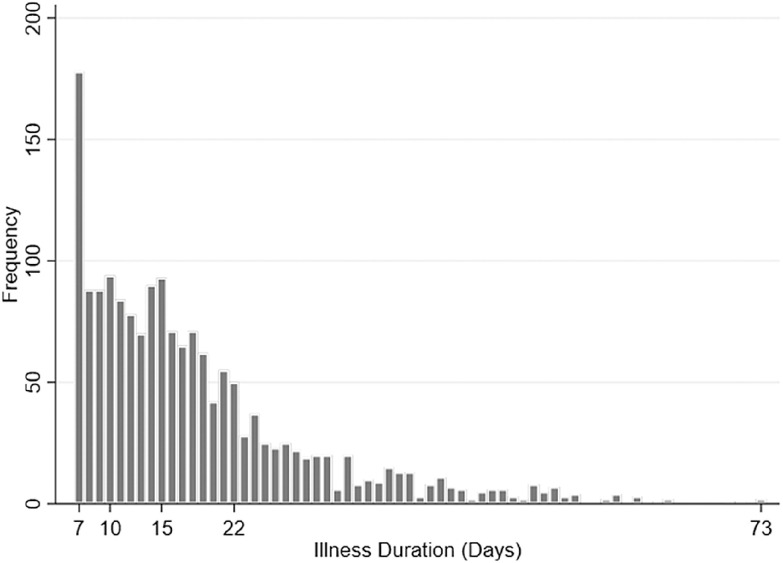
Frequency distribution of illness days among heathcare workers (HCWs). Study population: HCWs who reported at least 1 symptom and who had a positive PCR test within −2 and +30 days of symptom onset and were cleared to return to work by OHS from March 1 to June 1, 2020. Median, 15 days; interquartile range, 10–22 days; minimum, 7 days; maximum 73 days.

In total, 1,698 HCWs were away from work due to COVID-19 symptoms, had a positive PCR test within a month of symptom onset, and were cleared by OHS to return to work during our study period. Illness duration ranged from 7 to 73 days (median, 15 days, interquartile range [IQR], 10–2) (Fig. 1). Mean age was 43.91 years (standard deviation, 12.13) and the median time between symptom onset to PCR test was 4 days (IQR, 2–7).

In bivariate analyses, 5 symptoms were associated with longer illness duration at 2-sided *P* < .05 (Table 1). In a multivariable model with backward variable selection including these symptoms, and controlling for age, 4 symptoms were significantly associated with longer illness durations: shortness of breath (5.64 days; standard error [SE], 0.73), fever (2.94 days; SE, 0.74), sore throat (2.25 days; SE, 0.73), and diarrhea (2.14 days; SE, 0.76) (Table 1).

### PCR versus symptom analysis

In total, 3,971 HCWs reported symptoms of possible COVID-19 and had a PCR test within a month of symptom onset. Of those, 2,059 (51.9%) HCWs tested positive. Among those with positive tests, the most commonly reported symptoms were cough (n = 1,652, 80.2%) and fever (n = 1,287, 62.5%) (Table 2).

In bivariate analyses, 7 symptoms were significantly associated with the PCR result (Table 2). In a multivariable model with backward variable selection including these symptoms, 6 symptoms showed a significant association with the odds of a positive PCR test. Anosmia (odds ratio [OR], 3.17; 95% confidence interval [CI], 2.05–4.91), ageusia (OR, 2.34; 95% CI, 1.56–3.51), cough (OR, 1.93; 95% CI, 1.43–2.62), and fever (OR, 1.80; 95% CI, 1.33–2.45) were associated with higher odds of a positive PCR. Sore throat (OR, 0.53; 95% CI, 0.39–0.72) and diarrhea (OR, 0.67; 95% CI, 0.48–0.95) were associated with lower odds (Table 2).

### Antibody versus symptom analysis

In total, 1,472 HCWs reported symptoms of possible COVID-19 and had an IgG antibody test, of whom 815 (55.4%) had reactive IgG antibody results. Of those with reactive tests, the most commonly reported symptoms were also cough (n = 637, 78.2%) and fever (n = 490, 60.1%) (Table 3).

In bivariate analyses, 5 symptoms were significantly associated with the IgG antibody result (Table 3). In a multivariable model with backward variable selection including these symptoms, only 2 symptoms showed a significant association with the odds of a reactive IgG antibody result at *P* < .05. Ageusia was the only symptom associated with higher odds of IgG antibody-reactivity (OR, 3.81; 95% CI, 2.07–7.00), and sore throat was associated with lower odds (OR, 0.49; 95% CI, 0.30–0.81) (Table 3).

### PCR versus IgG antibody test results

In total, 1,635 HCWs received both PCR and IgG antibody tests (Table 4). Of the 776 HCWs who had been PCR positive, 721 (92.9%) went on to have reactive IgG antibody responses. Meanwhile, of the 859 HCWs who had been PCR negative, 95 (11.1%) had reactive IgG antibody tests.

**Table 4.** PCR vs. IgG Antibody Test Results^a^

	IgG Antibody Results		
	Reactive	Nonreactive	
PCR Result	No (%)	No (%)	Total
Positive	721 (92.9)	55 (7.1)	776
Negative	95 (11.1)	764 (88.9)	859
Total	816 (49.9)	819 (50.1)	1,635

Note. PCR, polymerase chain reaction; IgG, immunoglobulin G. Study population: healthcare workers (HCWs) who had both the PCR and IgG antibody tests from March 1 to June 12, 2020.

## Discussion

During spring 2020, COVID-19 had a profound impact on the work force of our large, urban, academic medical center and the communities we serve. Between March 1 and June 1, HCWs who had symptoms of COVID-19 and positive PCR tests were ill for a median of 15 days (IQR, 10–22). Because we excluded anyone who required hospitalization and those still away from work on June 1, the median duration for all HCWs is likely longer than we report here. A recent CDC study found that more than one-third of milder COVID-19 patients had not fully recovered within 14–21 days of symptom onset.^5^ This result is similar to our findings in HCWs. In our study, longer illness durations were associated with symptoms of fever, shortness of breath, sore throat, and diarrhea. Most notably, the presence of shortness of breath was associated with illnesses that were 5.64 days longer, which has not been demonstrated in other studies to our knowledge. Diarrhea has been associated with longer illnesses in inpatient settings.^3,4^

COVID-19 symptoms overlap with many other illnesses. This factor makes staffing even more difficult because HCWs with common symptoms require temporary removal from the work force. Of the symptomatic HCWs who had PCR testing, 51.9% tested positive. This proportion parallels the percentage of symptomatic HCWs with reactive IgG antibody tests (55.4%). Although it is impossible to know with certainty whether the reported symptoms actually derived from the event that caused seroconversion of IgG antibodies, the similarities in these numbers suggest that slightly more than 50% of HCWs who reported symptoms of possible COVID-19 actually had the disease during a period of high overall prevalence. This percentage is higher than that observed in similar studies, which have reported PCR positivity among 5%–15% of symptomatic HCWs.^7–9^ This difference is likely due to the higher prevalence of COVID-19 in the New York metropolitan area during spring 2020.^23^

We also identified the symptoms that are more likely to be associated with a positive PCR or reactive IgG antibody test. The presence of anosmia, ageusia, fever, or cough were all associated with higher odds of PCR positivity, and sore throat or diarrhea were associated with lower odds. This finding is consistent with several studies that found anosmia and ageusia to have higher odds of PCR positivity^7–12^ and a few reported associations with fever and cough.^7–10^ Although muscle aches and fatigue have also been reported to be associated with PCR-positivity,^7–10^ we did not find these associations despite the high prevalence of these symptoms in our study population (Table 2). Our study results also indicated that sore throat and diarrhea lowered the odds of PCR positivity. Other illnesses prevalent during the spring, such as seasonal allergies or upper respiratory infections, also commonly present with sore throat, could partially explain the lower odds. To our knowledge, no other study has reported lower odds of PCR positivity with sore throat or diarrhea.

Only ageusia had higher odds of IgG antibody reactivity, whereas sore throat was again associated with lower odds. This finding is similar to that of other studies of HCWs,^14,15^ though we did not find significant associations between IgG antibody reactivity and fever, cough, or anosmia, as others have reported.^13,15^ It is not immediately clear why there are differences in the symptoms associated with reactive IgG antibody tests compared to those associated with PCR positivity. However, the PCR and IgG antibody cohorts were different populations of HCWs. Fewer than one-third of HCWs included in our PCR-symptom analysis went on to receive IgG antibody tests by the time we ran the analysis, possibly because (1) people with confirmed COVID-19 were less likely to seek confirmation from an antibody test, (2) the timing of our study versus the availability of antibody tests, and/or (3) the need to wait 21 days from the onset of symptoms.

Among HCWs who had both tests, 92.9% of those who were PCR positive also had reactive IgG antibody tests. It is not clear how long IgG antibodies last after infection and what the impact of developing antibodies is on the risk of future infection. However, if antibodies offer any protection, the high rates of developing antibodies in HCWs would be beneficial to this group who interacts with many COVID-19–positive people. Some studies have found that all patients who were PCR positive went on to eventually develop antibodies,^24,25^ but others have found some discordance, as in our study.^12,26^ Possible reasons for discordance might include absent or diminished antibody production in some HCWs^27^ or that these HCWs either had not yet produced antibodies or that their antibody production had waned by the time of testing.^27,28^ Of those who were PCR negative, 11.1% went on to have reactive IgG antibody tests. Potential reasons for this discordance include (1) that the SARS-CoV-2 infection could have been asymptomatic or distinct from the symptom complex that triggered PCR testing,^29,30^ (2) a false-negative PCR result,^16–18^ or (3) a false-positive IgG antibody test.

Although our analyses included a large cohort of our HCW population, our study had several limitations that reflect the real-world nature of this pandemic study. Data capture was limited by reliance upon self-reporting by HCWs of symptoms and illness status to the OHS call center. We relied upon verbal reports for tests done outside of MMC. A surge in call volume exceeded existing infrastructure at the beginning of data capture, resulting in possibly incomplete acquisition of HCW data. The type of PCR tests used among testing locations varied, as did the time between symptom onset and PCR tests among the study population. Several symptoms were added midway through data collection based on evolving guidance from the CDC and NYSDOH. In addition, not all symptomatic HCWs were able to obtain PCR testing at the beginning of the pandemic due to limited supplies. More than 80% of symptomatic MMC HCWs were able to get a PCR test during our study period; however, it is possible that our results were biased by the institution’s decision to prioritize testing for the sickest HCWs when resources were limited.

We also recognize several limitations in our data analysis. First, because of the changing guidance from the CDC and NYSDOH with regard to COVID-19–related symptoms, some symptoms (eg, diarrhea, anosmia, and ageusia) were not being discretely captured until weeks into the study period. New York state’s peak of new daily cases occurred on April 14; however, several symptoms were added on April 17, when the state’s case load was already trending downward.^31^ This addition led to missing symptom data for some HCWs, which ultimately meant that they were dropped from models that included those symptoms. Thus, our results may have been biased toward those symptoms that were being captured when the prevalence of COVID-19 was at its highest from mid-March until mid-April and for which this study had the highest power to detect associations. Also, we were unable to delineate the specific durations of individual symptoms. Accordingly, our duration analysis is limited to extrapolation from the HCW’s overall illness length, as opposed to the duration of the symptom in question. The presence of shortness of breath at any point during an illness was associated with prolonged duration by 5.64 days; however, we were unable to determine whether that symptom lasted for the entirety of the illness.

COVID-19 has had a profound impact on the healthcare work force, not only because of the number of infected HCWs but also because of the amount of time that HCWs need to be away from work. The human resources service at our institution reports a 53% increase in sick hours used by HCWs between March 1 and June 1 in 2020 versus the same period in 2019. In our study, HCWs with milder cases of COVID-19 were ill for a median of just more than 2 weeks. Fever, shortness of breath, sore throat, and diarrhea were associated with longer illnesses. During a period of high prevalence, more than half of HCWs with COVID-19 symptoms tested positive for the disease. Our findings support the need for hospitals and occupational health departments to assume that HCWs with symptoms of COVID-19 will be away from work for ~2 weeks when making schedules and requesting surge staff. Occupational health departments could use these findings to help identify HCWs taking longer than average to recover and expedite referrals to COVID-19 clinics and specialists. The ability to capture and correlate symptoms with illness duration could ultimately lead to predictive modeling that would allow for anticipatory support to be put in place both for the hospital and the HCWs. Further study of how HCWs have been impacted by illness from COVID-19 is critical so we can maintain a robust response to our patients and provide answers to our HCWs, who are the backbone of the fight against this new pandemic.
